# Short-term exercise training improves cardiac function associated to a better antioxidant response and lower type 3 iodothyronine deiodinase activity after myocardial infarction

**DOI:** 10.1371/journal.pone.0222334

**Published:** 2019-09-12

**Authors:** Rafael Aguiar Marschner, Patrícia Banda, Simone Magagnin Wajner, Melissa Medeiros Markoski, Maximiliano Schaun, Alexandre Machado Lehnen

**Affiliations:** 1 Institute of Cardiology of Rio Grande do Sul/University Foundation of Cardiology, Porto Alegre, Rio Grande do Sul, Brazil; 2 Thyroid Division, Endocrinology Service, Hospital de Clínicas de Porto Alegre/Federal University of Rio Grande do Sul, Porto Alegre, Rio Grande do Sul, Brazil; 3 Federal University of Health Sciences of Porto Alegre, Porto Alegre, Rio Grande do Sul, Brazil; Scuola Superiore Sant’Anna, ITALY

## Abstract

**Aims:**

We assessed the effects of a short-term exercise training on cardiac function, oxidative stress markers, and type 3 iodothyronine deiodinase (D3) activity in cardiac tissue of spontaneously hypertensive rats (SHR) following experimental myocardial infarction (MI).

**Methods:**

Twenty-four SHR (aged 3 months) were allocated to 4 groups: sham+sedentary, sham+trained, MI+sedentary and MI+trained. MI was performed by permanent ligation of the coronary artery. Exercise training (treadmill) started 96 hours after MI and lasted for 4 weeks (~60% maximum effort, 4x/week and 40 min/day). Cardiac function (echocardiography), thioredoxin reductase (TRx), total carbonyl levels, among other oxidative stress markers and D3 activity were measured. A Generalized Estimating Equation was used, followed by Bonferroni’s test (p<0.05).

**Results:**

MI resulted in an increase in left ventricular mass (p = 0.002) with decreased cardiac output (~22.0%, p = 0.047) and decreased ejection fraction (~41%, p = 0.008) as well as an increase in the carbonyl levels (p = 0.001) and D3 activity (~33%, p<0.001). Exercise training resulted in a decrease in left ventricular mass, restored cardiac output (~34%, p = 0.048) and ejection fraction (~20%, p = 0.040), increased TRx (~85%, p = 0.007) and reduced carbonyl levels (p<0.001) and D3 activity (p<0.001).

**Conclusions:**

Our short-term exercise training helped reverse the effects of MI on cardiac function. These benefits seem to derive from a more efficient antioxidant response and lower D3 activity in cardiac tissue.

## Introduction

Myocardial infarction (MI) leads to changes in the oxidative metabolism of cardiomyocytes in response to ischemic injury, which arises from an imbalance between reactive oxygen species (ROS) production and antioxidant agents [1]. On the other hand, thyroid hormone homeostasis is regulated by the synchronized activity of iodothyronine deiodinases: types 1 and 2 catalyze the conversion of prohormone thyroxine (T4) into the biologically active triiodothyronine (T3); and type 3 iodothyronine deiodinase (D3) catalyzes the inactivation of T4 and T3 into inactive forms. Thus, increased D3 activity may lead to lower intracellular T3 levels and impaired cellular metabolism.

An increase in D3 was shown in a rat model of right ventricular hypertrophy due to pressure overload [2]. We also have found increased D3 activity after MI associated to imbalance in the redox state (pro-oxidative) [3]. MI-induced increase in D3 activity was associated with lower T3 concentration in cardiomyocytes, whereas plasma T3 levels were unchanged [4]. Therefore, long-term effects caused by increased D3 activity as a result of imbalance in the redox state appear to contribute to pathologic ventricular remodeling and lower cardiac function [5].

Regular exercise is well known to enhance cardiac function both as a preventive measure [6] and after acute MI [7]. This benefit may be associated with improved antioxidant response [8, 9]. In view of an increase in D3 activity following acute MI and that this scenario is associated to an altered redox state [10], our hypothesis is that exercise training can improve cardiac function and is associated with lower D3 activity due to improved redox balance. However, most studies assessing the benefits of exercise on cardiac function and antioxidant activities have investigated long-term training programs of 10–14 weeks or more [11, 12]. Thus, we investigated whether beneficial changes on cardiac function associated with lower D3 levels and improved oxidative stress balance can occur in response to a short-term exercise training or only after long-term training. Our study aimed to assess the effects of a short-term exercise training on cardiac function, oxidative stress markers, and D3 activity in spontaneously hypertensive rats (SHR) with acute MI. Secondarily, we evaluated the expression of hypoxia-inducible factor 1 (HIF-1) and vascular endothelial growth factor (VEGF) considering that HIF-1 is primarily involved in promoting angiogenesis [13], and that reduced HIF-1 levels in the myocardium may be associated with increased ROS [14].

## Materials and methods

Our study complied with the ethical principles of the National Institutes of Health guide for the care and use of Laboratory animals (NIH Publications No. 8023, revised 1978) and approved by the Research Ethics Committee at Institute of Cardiology of Rio Grande do Sul, Brazil (protocol #5113/15). All animals provided a standard rat chow and water *ad libitum*, and maintained in a controlled 12-h light/12-h dark cycle (6 am/6 pm) under 20–25°C conditions.

Considering that hypertension is a predictor of MI, increased D3 levels were found in a model of right ventricular hypertrophy due to pressure overload and systemic arterial hypertension may lead to pressure overload, we hypothesized that SHR is a convenient model because they present with hypertension (of spontaneous systemic development as seen in humans) [15]. Thus, twenty-four male SHR (aged 3 months) were allocated to 4 groups (n = 6/group): sham + sedentary, sham + trained, MI + sedentary and MI + trained.

### Blood pressure

**Fig 1:**
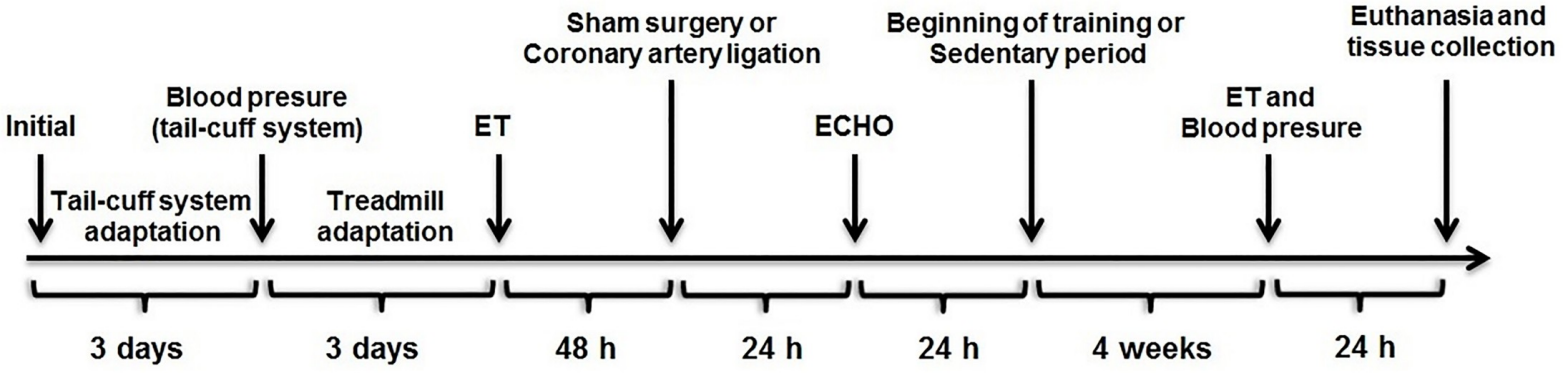
Study timeline. ET: maximum running speed in the exercise test.

BP was measured using a tail-cuff system (Model 229, IITC Life Science Inc.). One day after an adaptation period (3 days) in a restrainer for 15 minutes, the final measurement was made. Hemodynamic collections were performed before surgical procedures and post-protocol of exercise training or sedentary period—Fig 1.

### Myocardial infarction and sham surgery

Myocardial infarction was induced according to a procedure previously performed in our laboratory [6, 16]. Briefly, animals were placed in the dorsal decubitus position and anaesthetized with isoflurane (100mL, Cristália). After orotracheal intubation, animals were submitted to mechanical ventilation with a Harvard ventilator, Model 683 (Holliston, MA, USA). A surgical incision was made in the skin along the left sternal margin, and divulsion of the pectoral and transverse muscle was carried out. Thoracotomy was carried out in the second intercostal space, and the thorax was opened without exteriorization of the heart. The left anterior descending coronary artery was identified and occluded with a 6–0 mononylon suture between the left atrial appendage margin and the pulmonary artery. The thoracic cavity was then closed with a 5–0 mononylon thread, the muscles were repositioned and the skin sutured. All animals received analgesic after the surgical procedure (tramadol, 0.5mL/kg, intraperitoneal). Animals that were submitted to a similar surgical procedure, but without coronary artery ligation, were used as sham-operated.

### Echocardiography

After anesthesia with isoflurane (100mL, Cristália), the animals were left lateral decubitus (45°) to obtain the echocardiographic images. Analyses of left ventricle (LV) ejection fraction (LVEF), left ventricle shortening fraction (LVSF), and fractional area change (FAC) were conducted 48 hours after AMI (Fig 1). The infarction area was delimited taking into account the movement of LV walls, by the observation of longitudinal, apical and transversal views of the LV. Regions with systolic thickness under normal, as well as portions with paradoxal movement, were considered as infarcted. The infarcted area (%) was thus determined by the ratio of these regions by total area of LV walls [17].

These analyses were performed using an EnVisor (Philips, Andover, USA) echocardiograph with a 12 MHz transducer. All measures and calculations of heart function parameters followed the procedures described in the literature [18]. Furthermore, left ventricular mass index (LVMI) was calculated by ratio of left ventricular mass (mg)/body mass (g).

### Maximal exercise test

Maximum effort capacity was measured using the exercise test as used in our laboratory [19–21]. After an adaptation period (3 days) on the treadmill at a speed of 0.3 km/h (15 minutes), the maximal exercise test was then performed individually at an initial speed of 0.3 km/h with increments of 0.3 km/h every 3 minutes until voluntary exhaustion of the animal—Fig 1.

### Exercise training

The dynamic aerobic exercise training, prescribed based on maximal running speed of the ET, was performed at low-moderate intensity (~ 50% to 70% ET) for 40 minutes a day (7 pm to 8 pm), 4 days a week for 4 weeks. Thus, we used a gradual increase in speed from 0.6 (50% ET) to 1.2 km/hr (70% ET)—Table 1, as already performed in our laboratory [8].

**Table 1:** General characteristics of the experimental animals.

	SS		ST		IS		IT	
	Baseline	Final	Baseline	Final	Baseline	Final	Baseline	Final
Body mass (g)	277.6±11.6^a^	305.3±17.1^b^	272.5±13.3^a^	312.8±22.0^b^	283.2±26.4^a^	318.7±20.4^b^	280.7±15.7^a^	299.7±21.2^b^
ET (km/h)	1.23±0.27^a^	1.31±0.27^a^	1.25±0.23^a^	2.20±0.24^b^	1.35±0.16^a^	1.25±0.12^a^	1.20±0.35^a^	2.14±0.21^b^
LVM (g)	------	1.29±0.10^a^	------	1.32±0.10^a^	------	1.91±0.25^b^	------	1.48±0.21^c^
LVMI (mg/g)	------	4.23±0.26^a^	------	4.23±0.25^a^	------	6.01±0.89^b^	------	4.87±0.86^a^

SS: sham + sedentary; ST: sham + exercise training; IS: acute MI + sedentary; IT: acute MI + exercise training; ET: maximum running speed in the exercise test; LVM: left ventricular mass; LVMI: left ventricular hypertrophy index, ratio of left ventricular mass (mg)/body mass (g). Differences were analyzed using generalized estimation equations (GEE) with two factors (sham/acute MI, sedentary/trained and the interaction between these factors), followed by Bonferroni post-hoc (p<0.05). Same letters indicate no statistical significance.

### Euthanasia and tissue collection

The animals were anesthetized with vaporizer containing isoflurane (100mL, Cristália) and a decapitation procedure (by guillotine) was used as euthanize. After that, cardiac tissue was rapidly removed and frozen in liquid nitrogen before being stored (-80C) for subsequent analyses.

### Non-enzymatic antioxidant defenses

Reduced glutathione (GSH) levels were measured according to a standard method. Briefly, tissues were homogenized in the presence of 300 μL of 20 mM sodium phosphate and 140 mM KCl buffer, pH 7.4. Proteins were precipitated by adding sodium metaphosphoric acid for a final ratio of 1:1. Samples were centrifuged for 10 min at 7,000 g. Fifteen microliters of tissue preparation was incubated with an equal volume of phothaldialdehyde (1 mg/mL methanol) at room temperature for 15 min in the presence of 20 volumes (1:20, v/v) of 100 mM sodium phosphate buffer, pH 8.0, containing 5 mM EDTA. Fluorescence was measured using excitation and emission wave lengths of 350 nm and 420 nm, respectively. A calibration curve was generated using standard GSH (0.001–0.1 mM), and GSH concentrations were calculated as nmol/mg protein.

Total glutathione (tGS) and oxidized glutathione (GSSG) levels were determined using the enzymatic recycling method [22], with some modifications. Briefly, tissues were homogenized in 4 (w/v) volumes of a sulfosalicylic acid solution (11%) and Triton X-100 (0.11%) (1:1 ratio). After incubating for 5 min at 4°C with continuous shaking, the samples were centrifuged at 10,000 g for 10 min (4°C), and the supernatant was collected for analyses of glutathione levels. For GSSG measurement, 10 μL of the supernatant was added to 110 μL of a GSH masking buffer (100 mM phosphate buffer, 1 mM EDTA, 1.1% 2-vinylpyridine), pH 7.4, and incubated for 1 h at room temperature. The samples prepared for tGS and GSSG measurement were subjected to enzymatic analysis in a recycling buffer system containing 300 μM NADPH, 225 μM DTNB, 1.6 U/mL GR and 1.0 mM EDTA in 100 mM phosphate buffer (pH 7.4). The linear increase in absorbance at 405 nm over time was monitored using a microplate reader (SpectraMax M5, Molecular Devices, California, US). A standard curve was generated using known amounts of GSH (100 μM) and GSSG (3.47, 6.95, 13.89 uM).

### Determination of thioredoxin reductase (TRx) and peroxide reductase (GPx) activities

Thioredoxin reductase activity (TRx) was determined using the Thioredoxin Reductase Assay Kit (Sigma-Aldrich, St. Louis, MO, USA) according to the manufacturer’s instructions. Briefly, heart tissue was lysed with a buffer containing 150 mmol/L NaCl, 50 mmol/L Tris-HCl, 1% Triton X-100, 1% sodium dodecyl sulfate, 1% deoxycholate, 1 mmol/L NaF and 1 mmol/L EDTA, and protease inhibitors. After centrifugation at 14 000 × g for 10 minutes at 4°C, protein concentrations of supernatants were determined using the Bradford method, and protein was then incubated in 100 mmol/L of potassium phosphate with 10 mmol/L EDTA and 0.24 mmol/L NADPH with and without a TRx reductase inhibitor of all isoforms. The reaction was started by adding 5,5′-dithiobis(2-nitrobenzoic) acid and monitored spectrophotometrically at 412 nm. Enzymatic activity was quantified using the following equation: Unit/mL = A412nm/minute (thioredoxin reductase) × dil × vol/enzvol (dil = sample dilution factor; vol = volume of reaction in mL; enzvol = volume of enzyme in mL). Results were expressed as U/mg protein. Glutathione peroxide activity (GPx) was determined using the Glutathione Peroxidase Cellular Activity Assay Kit (Sigma-Aldrich, St. Louis, MO, USA) according to the manufacturer’s instructions. Briefly, enzyme activity was determined in crude homogenate of heart tissue by monitoring the NADPH disappearance at 340 nm. The specific activity was calculated as U/mg protein.

### Determination of tissue protein carbonyl content

Duplicate aliquots of 0.3 mg of protein homogenate of cardiac tissue samples were incubated with 500 μL of 10 mM 2.4-dinitrophenylhydrazine or 1.0 mL of 2 M HCl (blank tube). After 30 min, 250 μL of 50% trichloroacetic acid was added to the aliquots. The samples were subsequently centrifuged at 8000 g for 30 min to obtain the protein pellets, which were immediately washed with ethanol-ethyl acetate at a 1:1 (v/v) ratio. The final protein pellets were resuspended in 500 μL of 8 M urea buffer and incubated at 50°C for 90 min. The difference between the 2.4-dinitrophenylhydrazine-treated and HCl-treated samples (blank) was used to calculate the carbonyl content determined at 370 nm. Carbonyl content was calculated using the millimolar absorption coefficient of hydrazine (e370 nm = 21,000000.M^-1^.cm^-1^), and the results were expressed in nmol carbonyl/mg protein [23].

### D3 activity assay

D3 activity in tissue samples was determined using paper chromatography as previously described [24, 25]. Tissues were individually homogenized and sonicated with 10mMTris-HCl, 0.25 sucrose buffer (pH 7.5), and 10mM dithiothreitol (DTT). Protein concentration was quantified by Bradford assay using BSA as a standard. The homogenates were incubated for an hour with 200,000 cpm 125I-labeled T3, 2 nM T3, 20mM DTT, and 1mM propylthiouracil (PTU) in order to inhibit any D1 activity. The addition of 200nM of T3 completely abolished D3 activity in all samples. The reaction was stopped by adding 200 uL ethanol 95%, 50 uL NaOH (0.04 N), and 5mg PTU. Deiodination was determined based on the amount of 125I-3, 3’-T2 produced after separation of reaction products by paper chromatography. Results were expressed as the fraction of T2 counts minus the non specific deiodination (always < 1.5%), obtained with the saturating concentration of T3 (200 nM). D3 activity was expressed as fentomoles T3 per minute per milligram protein. The quantity of protein assayed was adjusted to ensure that < 30% of the substrate was consumed.

### ELISA studies

Cardiac tissue samples were centrifuged (1,000g; 20 min) and submitted to ELISA kits (HIF-1, Cusabio, MD, EUA—kit #CSB-E08540r and VEGF, Cusabio, MD, EUA—kit #CSB-E04757r). All measurements were carried out using four-parameter linear regression.

### Statistical analysis

Data normality was tested using Shapiro-Wilk. The values are shown as mean ± SD. A generalized estimating equation (GEE) with Bonferroni’s post-hoc was used to examine the effect of exercise training on AMI according to two factors (sedentary/exercise training, sham/AMI, and the interaction between them). A Student’s t-test for independent samples was performed to analyze data on MI size (p<0.05 for all tests).

## Results

### Cardiac changes in response to myocardial infarction model

Table 1 summarizes general characteristics of the experimental animals. Their body mass increased over time (p<0.001) regardless of their status (acute MI/Sham or exercise training/sedentary period). MI induction increased left ventricular mass (LVM) (SS vs. IS, p = 0.002) and thus left ventricular mass index (LVMI) (SS vs. IS, p = 0.016) (Table 1).

Table 2 summarizes hemodynamic and echocardiographic parameters of the animals studied. Acute MI resulted in reduced cardiac output (SS vs. IS: ~22.0%, p = 0.047), reduced left ventricular ejection fraction (LVEF) (SS vs. IS: 40.5%, p = 0.008) and reduced left ventricle shortening fraction (LVSF) (SS vs. IS, p<0.001), evidencing a reduction in cardiac function in response to the ischemic injury of acute MI. Additionally, the extent of MI can be evaluated by measuring the akinetic area of the left ventricle. There was no difference of the akinetic area in IS and IT groups (p = 0.892), suggesting the accuracy of MI procedure and homogeneity of our results.

**Table 2:** Hemodynamic and echocardiographic parameters.

	SS		ST		IS		IT	
	Baseline	Final	Baseline	Final	Baseline	Final	Baseline	Final
SBP (mmHg)	181.4±4.0^a^	184.5±5.1^a^	185.3±9.8^a^	162.7±2.8^b^	192.0±12.4^a^	191.8±6.2^a^	181.1±3.4^a^	154.4±5.4^b^
HR (bmp)	337.8±32.4^a^	323.7±18.35^a^	357.6±27.3^a^	280.0±8.0^b^	341.2±16.8^a^	330.5±22.3^a^	340.5±22.0^a^	289.8±12.1^b^
CO (ml/min)	68.7±17.8^a^	70.5±11.4^a^	69.0±15.9^a^	66.7±12.2^a^	53.6±10.7^b^	52.9±19.5^b^	49.5±18.8^b^	70.8±15.3^a^
LVEF (%)	56.0±10.0^a^	61.2±3.7^a^	57.1±6.0^a^	66.7±5.9^a^	33.3±6.6^b^	33.9±10.0^b^	34.0±11.2^b^	40.5±6.8^c^
LVSF	42.1±5.7^a^	42.6±4.2^a^	43.5±4.2^a^	46.2±5.6^a^	17.0±5.8^b^	17.5±4.8^b^	20.4±3.4^b.c^	24.6±9.3^c^
AA	------	------	------	------	39.5±6.6^a,b^	35.1±7.4^a,b^	40.3±5.1^a^	31.5±6.1^b^
FAC	46.8±6.2^a^	50.0±3.7^a^	47.5±4.9^a^	50.9±7.4^a^	25.8±2.7^b^	28.1±7.1^b^	24.4±7.9^b^	29.4±9.2^b^

SS: sham + sedentary; ST: sham + exercise training; IS: acute MI + sedentary; IT: acute MI + exercise training. SBP: systolic blood pressure; HR: resting heart rate; CO: cardiac output; LVEF: left ventricular ejection fraction; LVSF: left ventricle shortening fraction; FAC: fractional area change; AA: akinetic area. Differences were analyzed using generalized estimation equations (GEE) with two factors (sham/acute MI, sedentary/trained and the interaction between these factors), followed by Bonferroni post-hoc (p<0.05). Same letters indicate no statistical significance.

**Fig 2:**
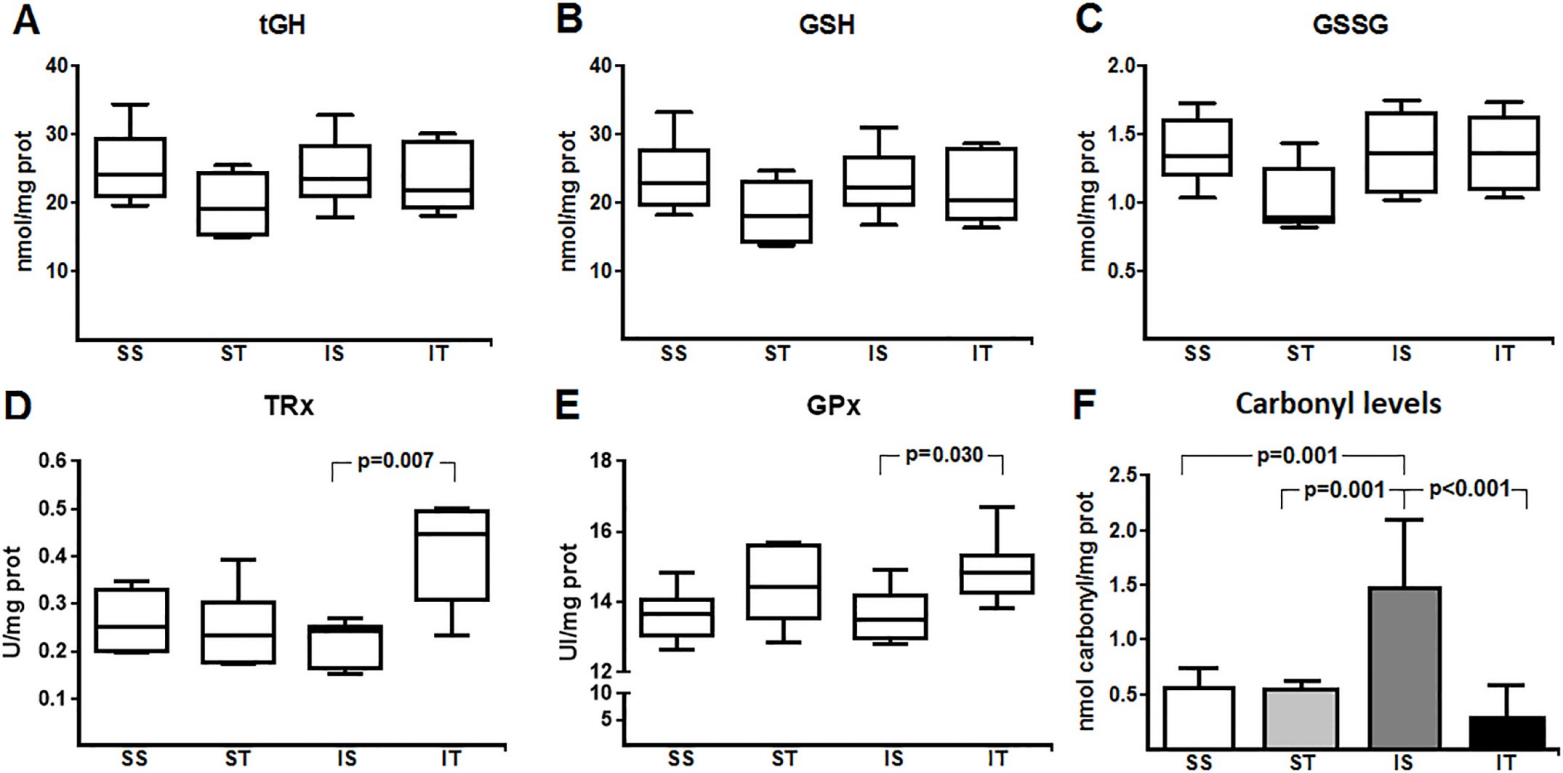
Redox state profile. SS: sham + sedentary; ST: sham + exercise training; IS: acute MI + sedentary; IT: acute MI + exercise training. Panel A: Total glutathione (tGH), Panel B: Reduced glutathione (GSH), Panel C: Oxidized glutathione (GSSH), Panel D: Thioredoxin reductase (TRx), Panel E: Glutathione peroxidase (GPx) and Panel F: Protein carbonyls levels. Differences were analyzed using generalized estimation equations (GEE) with two factors (sham/acute MI, sedentary/trained and the interaction between these factors), followed by Bonferroni post-hoc (p<0.05).

**Fig 3:**
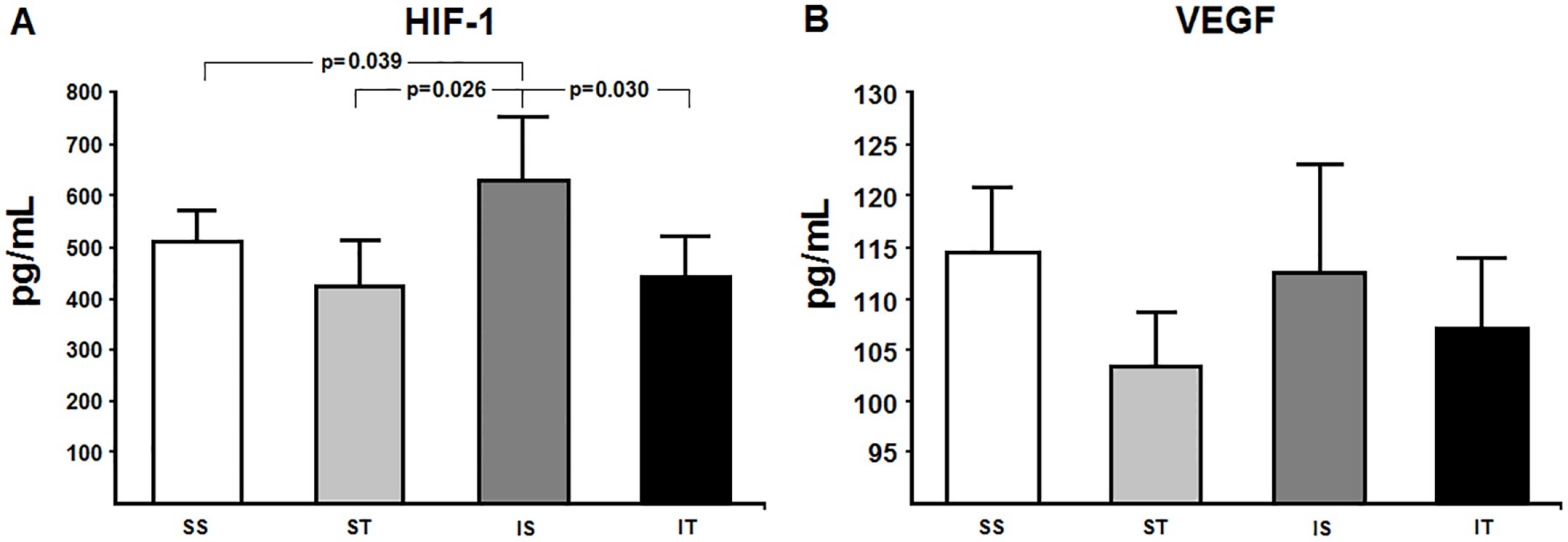
HIF-1 and VEGF expression levels. SS: sham + sedentary; ST: sham + exercise training; IS: acute MI + sedentary; IT: acute MI + exercise training. Panel A: Hypoxia inducible factor (HIF-1), p (interaction) = 0.032. Panel B: vascular endothelial growth factor (VEGF), though the interaction of these factors was not significant. Differences were analyzed using generalized estimation equations (GEE) with two factors (sham/acute MI, sedentary/trained and the interaction between these factors), followed by Bonferroni post-hoc (p<0.05).

**Fig 4:**
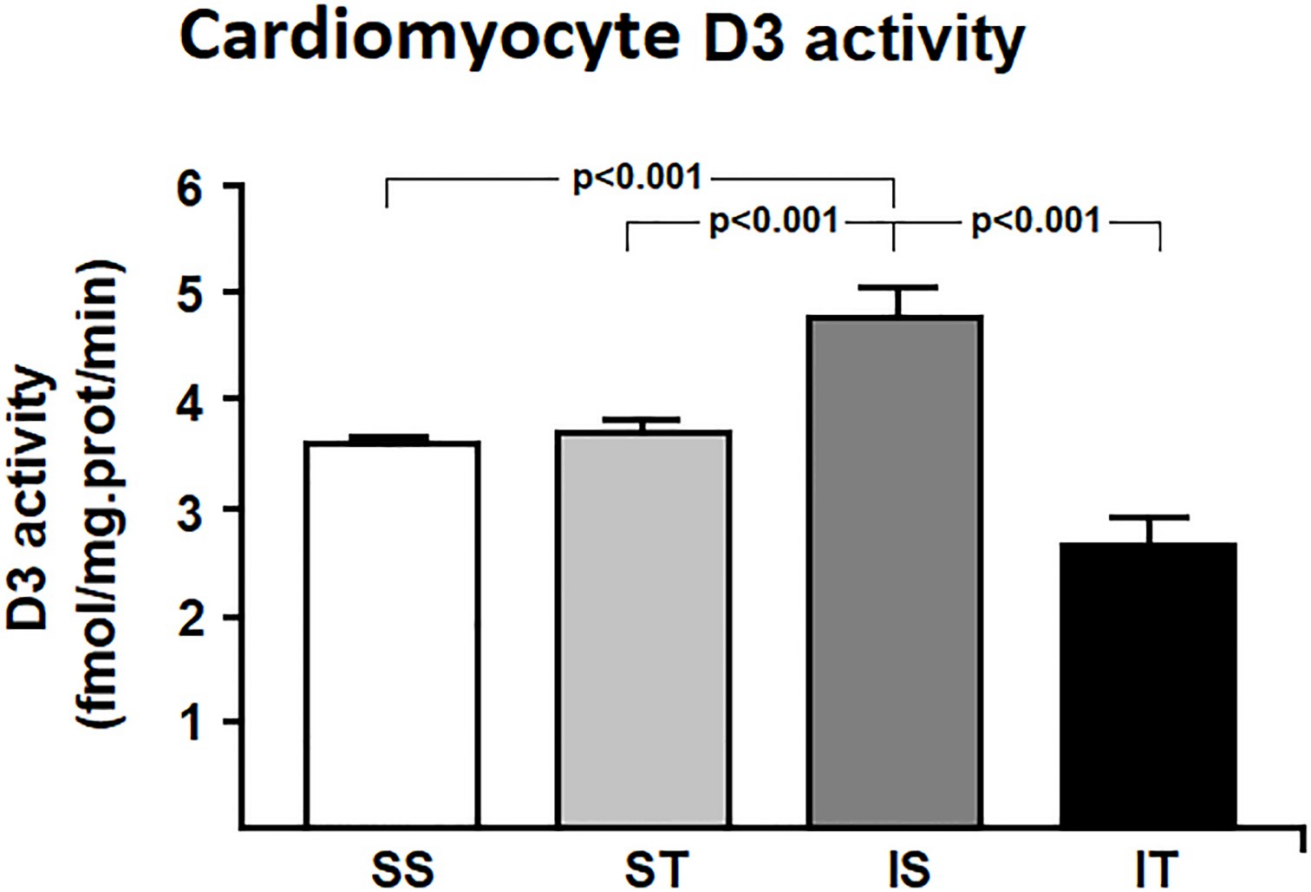
D3 activity in cardiac tissue samples. SS: sham + sedentary; ST: sham + exercise training; IS: acute MI + sedentary; IT: acute MI + exercise training. Differences were analyzed using generalized estimation equations (GEE) with two factors (sham/acute MI, sedentary/trained and the interaction between these factors), followed by Bonferroni post-hoc (p<0.05).

As for oxidative stress parameters (Fig 2), non-enzymatic antioxidants including tGH, GSH and GSSG did not show any changes either by MI or exercise (p = 0.260, p = 0.210, p = 0.070, respectively). However, carbonyl levels were high in response to acute MI (SS vs. IS: p = 0.001). In addition, acute MI led to an increase in HIF-1 levels when compared to the control group (SS vs. IS, p = 0.039) (Fig 3). Interestingly, there were no changes in the expression levels of VEGF (p = 0.427) (Fig 3). D3 activity increased in cardiac tissue in response to acute MI (SS vs. IS, 32.5%, p<0.001) (Fig 4).

### Cardiac changes in response to exercise training intervention

Maximum effort capacity, assessed by an exercise stress test, increased (p<0.001) in the trained groups (ST and IT) when compared to their controls (SS and SI, respectively) (Table 1). Interestingly, the same magnitude of improvement in the maximum stress test was seen in both groups (SS vs. ST: 76%, p<0.001; SI vs. IT: 79%, p<0.001). Yet, lower LVM and LVMI were found in the infarcted group undergoing training compared to its control (IS vs. IT, p<0.001) (Table 1).

The 4-week exercise protocol provided sufficient training to reduce systolic blood pressure (SBP) in the trained groups regardless of their status when compared to their controls (SS vs. ST: 118% and IS vs. IT: 19.4%, p<0.001 for both comparisons) (Table 2). Regarding the benefits for cardiac function, our protocol restored cardiac output in the infarcted animals (IS vs. IT, ~34%, p = 0.048), and improved LVEF (IS vs. TI, ~20%, p = 0.040) and LVSF (IS vs. IT, ~41%, p<0.001) (Table 2). Also, after the 4-week exercise training protocol, there was a ~22% reduction in the akinetic area compared to baseline data in IT group.

Our training protocol resulted in increased levels of enzymatic antioxidants, especially TRx by ~85% (IS vs. IT, p = 0.007) and GPx by ~10% (IS vs. IT, p = 0.030). In addition to increased enzymatic defenses, carbonyl levels (IS vs. IT, p<0.001) and HIF-1 levels were reduced in the infarcted animals undergoing training (IS vs. IT, p = 0.030), though VEGF levels did not change throughout the study (Fig 3). Finally, the exercise protocol decreased D3 activity in cardiac tissue in infarcted animals when compared to their controls (IS *vs*. IT, p<0.001).

### Correlation with D3 activity in cardiac tissue

**Fig 5:**
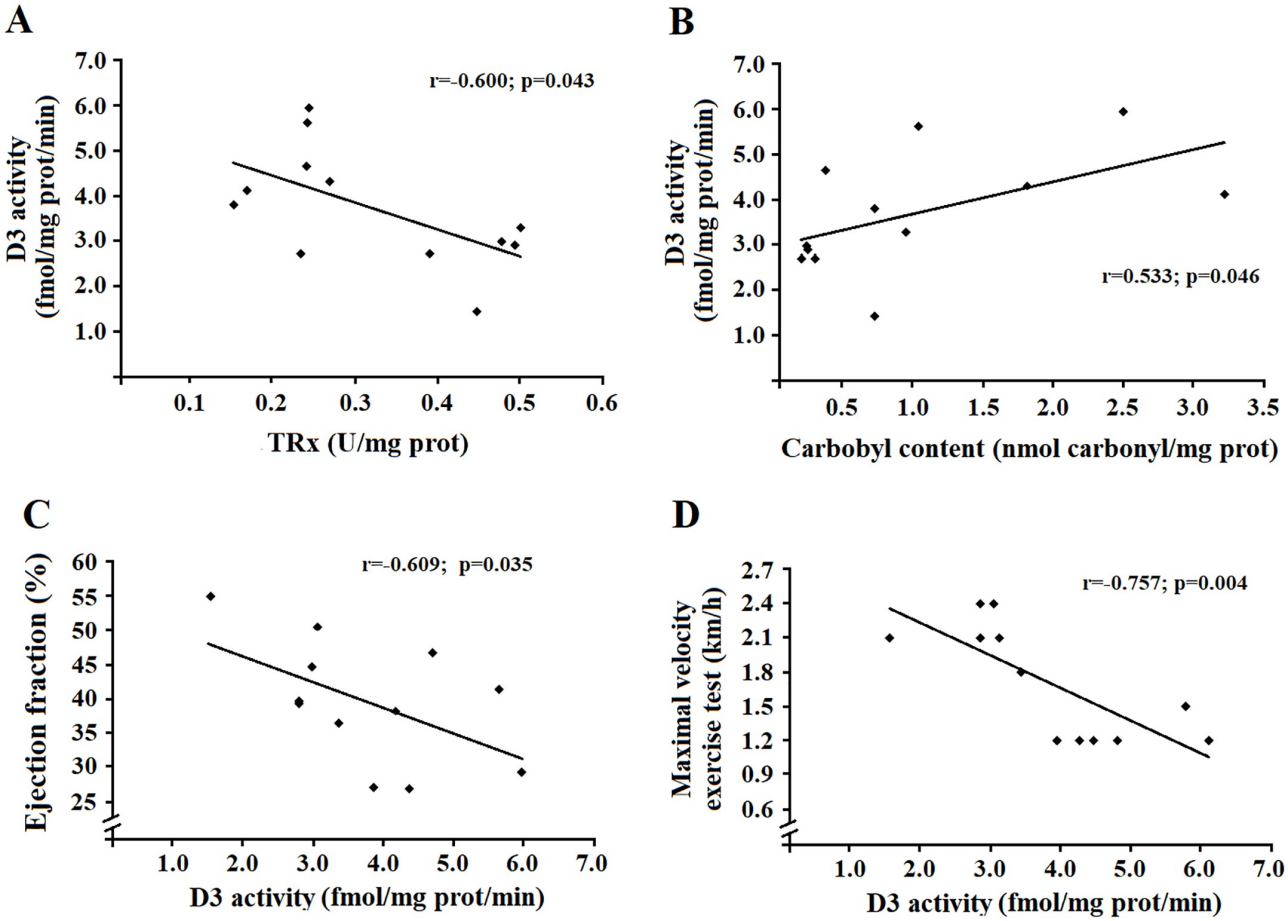
Correlations of D3 activity with oxidative stress parameters and cardiac performance (ejection fraction + maximal velocity exercise test). Panel A: correlation of thioredoxin reductase with D3 activity. Panel B: correlation of total protein carbonyl levels with D3 activity. Panel C: correlation of D3 activity with cardiac function (left ventricle ejection fraction). Panel D: correlation of D3 activity with performance in the maximal exercise stress test.

Fig 5 shows the correlations with D3 activity, changes in oxidative stress and cardiac function in the infarcted animals (IS and IT). D3 activity was inversely correlated with TRx levels (r = –0.600, p = 0.043) and directly correlated with carbonyl levels (r = 0.533, p = 0.046) (Fig 5). Cardiac function, measured by LVEF, was inversely correlated with D3 activity (r = –0.609, p = 0.035) and maximum effort capacity (r = –0.757, p = 0.004) (Fig 5).

## Discussion

Our study showed that a moderate-intensity exercise training protocol as short as of 4 weeks in duration can partially reverse the damaging effects of acute MI on cardiac function (cardiac output, LVEF, SBP, resting HR and maximum exercise capacity). The improvement in cardiac function seems to be associated with favorable redox balance change and a decrease in D3 activity in cardiac tissue. To the best of our knowledge, this is the first study investigating short-term exercise training and cardiac responses associated with changes in oxidative stress and D3 activity in SHR with experimentally-induced acute MI.

For an adequate assessment of the effects of a short-term exercise training on adverse events occurring in response to acute MI, we first have to examine our MI model. Following an experimentally-induced acute MI, SHR showed a reduction in cardiac output, LVEF, LVSF and ventricular fractional area change (FAC). These series of parameters is consistent with that assessed in other studies by our research group [6, 8, 16] and others as well [26, 27]. In addition, the reduction in cardiac function may be largely explained by increased protein oxidation (augmented carbonyl levels) and D3 activity in cardiac tissue sample of the infarcted animals [3]. With regard to hemodynamic parameters, SBP increased regardless of the animals’ status (acute MI or sham) showing that, in this experimental model, acute MI did not induce any hemodynamic changes.

Overall, the reduction in cardiac function in response to acute MI was reversed following a 4-week aerobic training protocol in our study, evaluated by cardiac output, LVEF, LVSF and FAC. As expected, our findings corroborate those reported in other studies. Evidences showed that animals with acute MI showed preserved cardiac function and improved LVSF upon completion of an exercise training protocol [28]. Regarding hemodynamic parameters, our training protocol resulted in a reduction of SBP and resting HR. These findings are consistent with a long-term aerobic training protocol (13 weeks in duration) in SHR that observed a reduction in SBP and resting HR in the trained group [29]. Similarly, low- or high-intensity exercise training has favorable hemodynamic effects in SHR [30]. We found in our study that SBP values show improvements with short-term protocol similar to those seen with longer protocols (13 weeks in duration).

Exercise training generates intermittent hypoxia in tissues due to increased cellular demand and increased ROS production during and after a training session [31, 32]. Enzymatic antioxidant responses are activated to counteract redox state changes in the tissue environment, with the consumption of non-enzymatic antioxidant reserve [33]. In long term, the antioxidant capacity of cardiac tissues is enhanced and acts as a protective factor in a state of redox imbalance [34]. We assessed the levels of tGH, GSH and GSSG in cardiac tissue to assess non-enzymatic antioxidant responses and redox state changes since GSH is a substrate for GPx that regulates the levels of hydrogen peroxide (H_2_O_2_) [35, 36]. There were no changes in tGH, GSH and GSSG levels in our study. However, we found an increase in GPx activity (by ~10%) in the IT group, even 28 days later. This finding suggests that our training protocol improved the effective of certain antioxidants such as GPx since the rate of GSH consumption and GSSG production remained the same. At the same time, TRx levels increased by ~85%, which may have contributed to a more effective redox balance and consequently a reduction in the extent of oxidative damage to cellular components. This assumption is supported by the finding of similar carbonyl levels in the IT and sham groups in our study.

Low levels of TRx have been also associated with left ventricular hypertrophy [37]. We found both increased TRx levels and lower LVMI in the IT group. This could be explained by TRx action inhibiting *cyclin D2* gene whose function is associated with left ventricular hypertrophy [38, 39].

Our findings also showed increased expression levels of HIF-1 in response to acute MI, even at a late assessment (4 weeks following ischemic injury), and unchanged VEGF levels. Increased HIF-1 levels may be more related to redox balance rather than VEGF transcription [40]. This assumption is supported in the literature [14]. The authors reported that, in a state of myocardial infarction/reperfusion, redox imbalance activates apoptotic pathways. In contrast, an increase in antioxidants favorably affects redox balance by reducing the expression of HIF-1. It seems reasonable to assume that lower expression of HIF-1 seen in the IT group may have resulted from increased antioxidant response (GPx and TRx) with ensuing reduction in carbonyl levels and inhibition of apoptotic pathways in myocardial tissue. Yet, two different 6-week swimming training protocols were investigated: moderate-intensity and high-intensity interval [41]. They found improved redox balance and lower HIF-1 levels in cardiac tissue of rats subjected to MI who underwent both training protocols. It corroborates our findings that lower HIF-1 levels may be a result of redox balance. On the other hand, VEGF levels remained unchanged in the groups studied. A possible explanation is that this protein is acutely increased during the ischemic process [11] but not in a later phase of MI [42] as assessed in our study.

Finally, changes in the redox state are known to induce D3 activity in disease, leading to several alterations in thyroid hormone metabolism [10, 43]. Studies after MI showed that increased D3 activity is associated with a 50% drop in T3 levels in heart tissue, reflecting in changes in the left ventricular geometry and remodeling [5, 44]. In line with others, our work showed induced post-MI D3 activity. Interestingly, we observed a reduction on D3 activity even with only four weeks of exercise training. It is possible to consider that the increase in the antioxidant defenses modulate the levels of D3 in the heart also in this circunstances. This is confirmed by the positive correlation of D3 with carbonyls and the negative correlation with TRx (Fig 5). These findings are in line with other studies, that demonstrated increased antioxidant defenses after MI in the presence of antioxidant medication, rebalancing the redox status, preventing changes in thyroid metabolism and improving cardiac function [3].

Considering the importance of thyroid hormones in cardiac function, several approaches tried to attenuate the deleterious effects of T3 inactivation [43]. In this way, exercise training becomes an excellent strategy, considering its benefits linked to thyroid metabolism [45, 46]. Our work demonstrated that exercise training was efficient to maintain cardiac function after MI, mediated by changes in D3 activity. It is suggested that dysfunctions in thyroid hormone levels are involved in the process of cardiac hypertrophy [4], changes in metabolism of thyroid hormones being the main cause leading to process of pathological remodeling [47, 48]. We also showed a negative correlation of D3 with the maximum effort capacity (Fig 5). These results are in agreement with the literature [4] that described an association between increased D3 activity and reduction in T3 concentrations in cardiac tissue samples, modifying left ventricular structure and function. To the best of our knowledge, this study was the first to use exercise training to correct the redox state and reduce D3 activity, resulting to improvements in the cardiac function. Interestingly, the results obtained with this approach are similar to studies that used antioxidant treatments to correct D3 function [3, 49].

In humans, an association of increased D3 activity—a key feature of low T3 syndrome—with decreased cardiac function in response to MI has been reported [50]. This is due to the fact that increased activity of D3 may account for reduced thyroid hormone action since this enzyme is responsible for inactivating intracellular T3 leading to changes in cardiac function. Studies involving human subjects have shown that therapies capable of restoring antioxidant defenses are excellent approaches to reestablish T3 biological activity [51]. Our study suggests that exercise training can effectively maintain redox balance and consequently reduce the extent of oxidative damage to myocardium. Thus, after MI individuals should participate in a cardiac rehabilitation program including regular aerobic exercise to improve their cardiac function, in according our results, by lowering D3 activity.

This study has some limitations. A major limitation is that we did not measure circulating T3 as well as myocardial tissue T3 levels. Post-MI rats exhibited lower T3 levels and they were back to normal and similar to those observed in control rats after receiving antioxidant therapy (N-acetylcysteine) [3]. Thus, as exercise training can improve antioxidant defenses, it would be interesting to compare T3 and T4 levels as well myocardial tissue T3 levels in response to exercise.

In conclusion, moderate-intensity aerobic training for a short period of time (4 weeks) was sufficient to partially reverse the harmful effects of acute MI. These benefits are associated with rebalancing the redox status and reducing D3 activity. Thus, regular physical exercise for a short period of time may have potential clinical implications in helping improve cardiovascular function following an acute MI.

## Supporting information

S1 Table Database Rafael Marschner.xls. (XLS)
